# Predictors of mortality and treatment success of multi-drug resistant and Rifampicin resistant tuberculosis in Zimbabwe: a retrospective cohort analysis of patients initiated on treatment during 2010 to 2015

**DOI:** 10.11604/pamj.2021.39.128.27726

**Published:** 2021-06-15

**Authors:** Ronnie Matambo, George Nyandoro, Charles Sandy, Tendai Nkomo, Shungu Mutero-Munyati, Sungano Mharakurwa, Elliot Chikaka, Mkhokheli Ngwenya, Gilchriste Ndongwe, Vongai Mildred Pepukai

**Affiliations:** 1 Biomedical Research and Training Institute, Harare, Zimbabwe,; 2 Community Medicine, College of Health Sciences, University of Zimbabwe, Harare, Zimbabwe,; 3 AIDS and Tuberculosis Department, Ministry of Health and Child Care, Harare, Zimbabwe,; 4 College of Health, Agriculture and Natural Sciences, Africa University, Mutare, Zimbabwe,; 5 World Health Organisation, Zimbabwe Country Office, Harare, Zimbabwe,; 6 Zimbabwe Evidence Informed Policy Network, Harare, Zimbabwe

**Keywords:** Predictors, mortality, treatment success, rifampicin-resistant tuberculosis, ART monitoring, TB/HIV co-infections

## Abstract

**Introduction:**

Zimbabwe is one of the 30 countries globally with a high burden of multidrug-resistant TB or rifampicin-resistant TB. The World Health Organization recommended that patients diagnosed with multidrug-resistant TB be treated with 20-24 month standardized second-line drugs since 2010. However, factors associated with mortality and treatment success have not been systematically evaluated in Zimbabwe. The Objective of the study was to assess factors associated with Mortality and treatment success among multidrug-resistant-TB patients registered and treated under the National Tuberculosis programme in Zimbabwe.

**Methods:**

the study was conducted using secondary data routinely collected from the National tuberculosis (TB) programme. Categorical variables were summarised using frequencies and a generalized linear model with a log-link function and a Poisson distribution was used to assess factors associated with mortality and treatment success. The level of significance was set at P-Value < 0.05.

**Results:**

patient antiretroviral therapy (ART) status was a significant associated factor of treatment success or failure (RRR = 3.92, p < 0.001). Patients who were not on ART had a high risk of death by 3.92 times compared to patients who were on ART. In the age groups 45 - 54 years (relative risk ratios (RRR) = 1.41, p = 0.048), the risk of death was increased by 1.41 times compared to other age groups. Patients aged 55 years and above (RRR = 1.55, p = 0.017), had a risk of dying increased by 1.55 times compared to other age groups. Diagnosis time duration of 8 - 30 days (RRR = 0.62, p = 0.022) was found to be protective, a shorter diagnosis time duration between 8 to 30 days reduced the risk of TB deaths by 0.62 times compared to longer periods. Missed TB doses of > 10% (RRR = 2.03, p < 0.001) increased the risk of MDR/RR-TB deaths by 2.03 times compared to missing TB doses of ≤ 10%.

**Conclusion:**

not being on ART when HIV positive was a major significant predictor of mortality. Improving ART uptake among those ART-naïve and strategies aimed at improving treatment adherence are important in improving treatment success rates.

## Introduction

There are an estimated 480,000 incident multidrug-resistant tuberculosis (MDR-TB) cases globally which were diagnosed among pulmonary TB (PTB) patients in 2013 [1]. An estimated 210 000 of the 1.5 million TB deaths globally in 2013 were due to MDR-TB [1]. Globally, cases of MDR/RR-TB continue to increase from 450,00 in 2012 to 480,00 in 2013 [2] MDR-TB is now considered a global public health emergency and poses a threat to global TB control programmes [3]. The pandemic is aggravated mostly in resource limited and low-income countries due to inadequate availability of prompt diagnostic and treatment measures [4]. Wells *et al*. described HIV/TB co-infection as the “Perfect storm” [5]. The World Health Organization (WHO) declared TB a “global emergency” [6]. There are some factors that have been described as associated with high mortality in tuberculosis Cases. These are TB drug-resistance [7], HIV infection [8-10], presence of diabetes mellitus [11], malignancy [12], alcohol abuse and malnutrition [13]. Treatment of MDR-TB requires the use of expensive and toxic second line anti-tubercular drugs given for a longer duration which often results in decreased compliance and success rates [14, 15]. Multi-drug resistant tuberculosis is mainly related to factors resulting from poor drug supply management, inappropriate guidelines none adherence to drug regimen, improper drug storage conditions, wrong dose or combination, and lack of patient education [16, 17]. According to 2016 WHO report, more than half a million people were newly eligible for MDR-TB treatment [18].

In 2010, WHO recommended directly observed treatment, short-course (DOTS) with 20-24 months standardized second-line drug (SLDs) regimens for treatment of MDR/RR-TB patients in low to middle income settings [19]. Zimbabwe is located in southern Africa and is among the 14 high burden countries (HBCs) with a triple burden of TB, TB/HIV and MDR-TB [20]. In 2017, Zimbabwe had an estimated 37,000 incident TB patients and 8,300 TB-associated deaths [20]. An estimated 1,300 MDR/RR-TB patients in the country in the same year with a prevalence of 4.6% and 14% among new and previously treated TB patients respectively was reported [20]. WHO estimated that less than 40% of the MDR/RR-TB patients were diagnosed and put on treatment in Zimbabwe in 2017 [21]. In the same report, among those initiated on treatment, more than 50% had unfavourable treatment outcomes. There has been no systematic assessment of predictors of Mortality and treatment success of MDR/RR-TB patients treated under the Zimbabwe National Trade Policy (NTP) nor has there been assessment of the individual and programmatic characteristics associated with Mortality and treatment success. Knowledge on predictors of mortality and treatment success among MDR/RR-TB patients can guide the NTP to make informed decisions on policies and strategies aimed at improving patient care for subsequent MDR/RR-TB patient cohorts. We therefore conducted a study aimed at assessing factors associated with mortality and treatment success among patients initiated on MDR/RR-TB treatment under the Zimbabwe NTP between 2010 and 2015.

## Methods

**Study design:** a retrospective study using secondary data routinely collected within the Zimbabwe NTP was conducted between January and December 2018.

**General setting:** there is an estimated 17 million people residing in Zimbabwe according to the Zimbabwe population census of 2012 [22]. Bordering Zimbabwe is Mozambique to the east, South Africa to the South, Zambia to the North and Botswana to the South West. There is a total of ten provinces in Zimbabwe of which two are Metropolitan provinces (Harare, the capital city and Bulawayo, the second largest city). These ten provinces are made up of 62 Districts of the country. There are four levels that make up the country´s public healthcare referral system in the country: 1) the quaternary level constituting six central hospitals located in the two metropolitan provinces; 2) the tertiary level consisting eight provincial hospitals which are the highest referral hospitals providing selected basic medical specialties for the eight rural provinces; 3) the secondary level constituting at least one district and or general hospital per district and last; 4) the primary care level consisting of rural and urban healthcare facilities that provide primary health care services.

In Zimbabwe, public healthcare facilities provide TB diagnosis, treatment and care and these services are integrated with general health services. Prior to 2013, only previously treated sputum positive pulmonary TB patients and MDR-TB contacts were considered as presumptive MDR-TB patients and evaluated for MDR-TB. Their sputum specimens were subjected to either phenotypic (culture and drug susceptibility testing (CDST)) or genotypic (MTB/Rif assay) testing. From 2013 onwards, Xpert MTB/Rif assay was used upfront for diagnosis of TB and rifampicin resistance in MDR-TB high risk groups. In all MDR/RR-TB patients, the remainder of the two collected sputum specimens is sent to one of the country´s two national reference laboratories for CDST in order to assess drug susceptibility to all the first line drugs. On registration at district hospital, the patient is notified to the NTP and a patient-held DR-TB treatment card is issued.

During the study period, the WHO recommended standardised DOTS-Plus regimen be used for management of MDR/RR-TB patients. The duration of treatment was at least 20 months with a minimum of six months (and 4 months after culture conversion) in the intensive phase and 14 months of the continuation phase. Oral drugs namely levofloxacin, pyrazinamide, cycloserine and ethambutol were given both during the intensive and continuation phases. The injectable kanamycin was provided six days a week during intensive phase. Treatment dosages were dispensed based on patient weight.

Patients are also offered provider-initiated HIV testing services in the MDR-TB pre-treatment phase and those found to be HIV positive are assessed for initiation on antiretroviral therapy (ART) and cotrimoxazole preventive therapy (CPT). As per the national guidelines in use during the study period, they were initiated on a fixed-dose combination once-daily pill of Tenofovir v+ Lamuvidine (or Emtricitabine) + Efavirenz (TDF+3TC (or FTC) + EFV) as the preferred first-line ART regimen among adult PLHIV and abacavir + lamuvidine+efavirenz (or Lopinavir/r) (ABC + 3TC + EFV (or Lop/r)) as the preferred first-line ART regimen in children living with HIV [23].

**Study population:** all MDR/RR-TB patients initiated on treatment between 2010 and 2015 under the Zimbabwe NTP and continued their treatment at either district hospitals or urban polyclinics were included in the study. Those patients who were referred to primary health facilities for DOTS-Plus treatment were excluded due to resource and time constraints in travelling to all primary healthcare facilities to collect their socio-demographic and clinical details.

**Data variables, sources of data and data collection:** patient demographic and clinical data were extracted from the health facility DOT register, individual patient clinical notes and the district DR-TB register using a structured proforma.Data extraction was done by District TB coordinators of the respective districts following training by the principal investigator.

**Data entry and analysis:** data were double entered and validated using EpiData entry software (EpiData Association, Odense, Denmark). Data were analysed using EpiData analysis (version 2.2.2.182, EpiData Association, Odense, Denmark) and Stata (version 12.0 STATA Corp., College, TX, USA). Categorical variables such as MDR/RR-TB deaths were summarized using numbers and percentages whilst medians (interquartile range (IQR) were calculated for skewed continuous data such as age and weight at treatment initiation. Unadjusted and multivariate-adjusted relative risks were calculated to obtain factors associated with “death” using univariate and multivariate generalized linear model with a log-link and binomial distribution or alternatively a poisson distribution with robust error variances if the model failed to converge. Potential factors with a p = 0.25 were included in the multivariate-adjusted regression model. A p-value < 0.05 was considered statistically significant.

## Results

### Demographics and clinical characteristics

**Figure 1:**
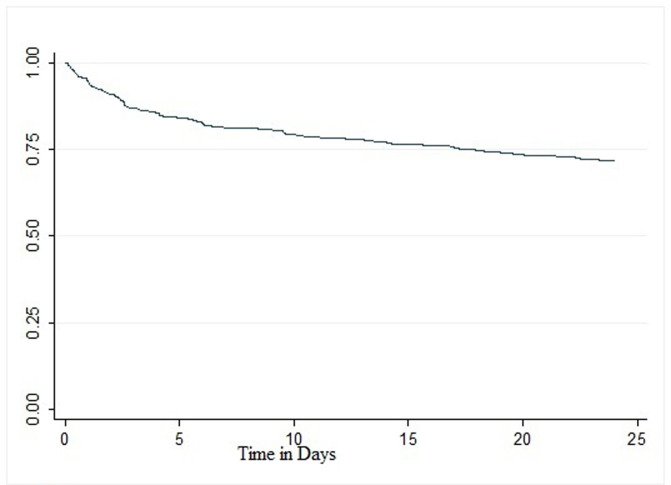
cumulative survival of MDR/RR-TB patients over time in days

The study enrolled 473 participants shown in Table 1 below of which 241 (51.0%) were females and 230 (48.6%) were males. Most participants were in the age category of between 25 to 34 years, 169 (35.7%) followed by 35 to 44 years, 149 (31.5%). The median age was 34 years with an interquartile range between 29 to 42 years. Most patients were married 202 (42.7%) followed by those who were single 143 (30.2%). Clinical findings amongst patients included in this study: show that 352 (74.4%) patients were HIV positive, 101 (21.4%) were HIV negative, 5 (1.1%) were not tested, whilst 15 (3.2%) were Missing. About 321 (91.2%) HIV positive patients were on ART, 18 (5.1%) were not whilst 13 (3.7%) were not recorded. Isoniazid drug susceptibility test (DRT) showed a relatively higher proportion of resistance among patients, 36.4% followed by Streptomycin, 14.0% and Ethambutol, 13.7%. This resistance may be linked to the observed adherence levels; DR TB treatment was completed by 77.2% of the clients on treatment whilst ART treatment was completed by 61.5% of the ART clients. The Kaplan - Meier graph (Figure 1) estimates survival at every time point in days, this analysis does not only show occurrence of deaths but, also in the time intervals in days the deaths occur. The cumulative probability of MDR/RR-TB patients surviving deaths decreases over time.

**Table 1:** demographic and clinical characteristics

Demographics characteristic		N (%)
Total		473(100%)
Gender	Male	230(48.6%)
	Female	241(51.0%)
	Missing	2(0.4%)
Age categories	<5 years	2(0.4%)
	5-14 years	10(2.1%)
	15-24 years	56(12.0%)
	25-34 years	169(36.2%)
	35-44 years	149(31.9%)
	45-54 years	47(10.1%)
	55 + years	34(7.3%)
Marital status	Married	202(42.7%)
	Single	243(30.2%)
	Widowed	44(9.3%)
	Divorced	25(5.3%)
	Missing	59(12.5%)
HIV status	Positive	352(74.4%)
	Negative	101(21.4%)
	Unknown/Untested	5(1.1%)
	Missing	15(3.2%)
ART status	On ART	321(91.2%)
	Not on ART	18(5.1%)
	Not recorded	13(3.7%)
Type of TB	New	257(54.3%)
	Retreatment after loss to follow-up	19(4.0%)
	Retreatment after failure	116(24.5%)
	Relapse	81(17.1%)
Treatment outcome	Cured	140(29.6%)
	Treated completely	149(31.5%)
	Died	125(26.4%)
	failed	4(0.9%)
	Loss to follow-up	39(8.3%)
	Not evaluated	16(3.4%)
Adherence to DR TB treatment	Never completed	83(17.5%)
	Completed	365(77.2%)
	Not sure	02(0.4%)
	Not recorded	23(4.9%)
Adherence to ART treatment	Never completed	29(6.1%)
	Completed	291(61.5%)
	Not recorded	153(32.3%)
Isoniazid DST pattern	Sensitive	35(7.4%)
	Resistant	172(36.4%)
	Not recorded	266(56.2%)
Ethambutol DST pattern	Sensitive	104(22.0%)
	Resistant	65(13.7%)
	Not recorded	304(64.3%)
Streptomycin DST pattern	Sensitive	99((21.0%)
	Resistant	66(14.0%)
	Not recorded	308(65.0%)

**Figure 2:**
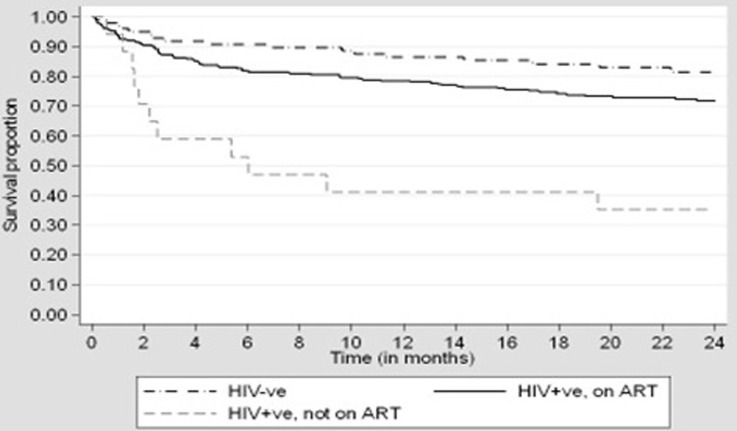
Kaplan-Meier survival estimates among patients registered and commenced on MDR/RR-TB treatment in Zimbabwe stratified by HIV and ART status

The Kaplan-Meier graph (Figure 2) show Survival estimates among patients registered and commenced on MDR/RR-TB treatment in Zimbabwe stratified by HIV and ART status. Most MDR/RR-TB deaths in patients who are HIV positive but not on ART occurred in the first 3 months upon treatment initiation. Further analysis using simple binomial regression modelling to test for factors associated with mortality are shown in Table 2. These factors are shown at the level of their single effect before adjusting them with other factors in the next modelling stage. The associations are shown in form of relative risk ratios (RRR) and the respective level of significance (Table 2). In the age groups 45 - 54 years (RRR = 1.41, p = 0.048), the risk of death was increased by 1.41 times compared to other age groups. Patients aged 55 years and above (RRR = 1.55, p = 0.017), had a risk of dying increased by 1.55 times compared to other age groups. Diagnosis time duration of 8 - 30 days (RRR = 0.62, p = 0.022) was found to be protective, a shorter diagnosis time duration between 8 to 30 days reduced the risk of TB deaths by 0.62 times compared to longer periods. Missed TB doses of > 10% (RRR = 2.03, p < 0.001) increased the risk of MDR/RR-TB deaths by 2.03 times compared to missing TB doses of = 10%;

**Table 2:** simple binomial regression modelling: showing significant factors associated with TB deaths

Outcome		RRR	Std. Err.	z	P>z	95% Conf Interval
**Female**		0.95	0.11	-0.41	0.685	0.76-1.2
**Age group**	≤24	0.86	0.18	-0.75	0.454	0.57-1.29
	35-44	1.06	0.15	0.4	0.69	0.8-1.41
	*45-54	1.41	0.25	1.97	0.048	1-2
	*55+	1.55	0.28	2.38	0.017	1.08-2.22
	Not recorded	0.92	0.54	-0.14	0.892	0.29-2.91
**Marriage status**	single	1.21	0.17	1.42	0.155	0.93-1.58
	widowed	1.36	0.25	1.66	0.097	0.95-1.95
	divorced	1.14	0.3	0.49	0.623	0.68-1.91
**Retreatment after**	loss to follow	0.92	0.28	-0.27	0.786	0.5-1.69
	failure	0.92	0.13	-0.55	0.585	0.7-1.22
	Relapse	0.95	0.15	-0.29	0.774	0.7-1.31
**Diagnosis time**	*8-30 days	0.62	0.13	-2.3	0.022	0.41-0.93
	31-90 days	0.8	0.2	-0.89	0.372	0.49-1.31
	>90 days	0.8	0.2	-0.89	0.372	0.49-1.31
	Not recorded	0.87	0.17	-0.7	0.482	0.59-1.28
**Isoniazid therapy**	resistant	2.2	0.94	1.83	0.067	0.95-5.1
**Ethambutol therapy**	resistant	0.8	0.21	-0.85	0.395	0.48-1.34
**Streptomycin therapy**	resistant	0.7	0.2	-1.26	0.206	0.4-1.22
**Culture conversion period**	> 6months	0.97	0.37	-0.07	0.944	0.46-2.05
	*Not recorded	2.96	0.38	8.42	0	2.3-3.8
**Missed TB doses**	≤10% missed	1.1	0.23	0.47	0.639	0.73-1.67
	*>10% missed	2.03	0.23	6.24	0	1.62-2.53
	Not recorded	1	(omitted)			
**Weight category**	≥50kg	1.04	0.16	0.23	0.822	0.76-1.41
**HIV status**	*HIV+ve	1.57	0.28	2.57	0.01	1.11-2.22
	*HIV unknown	2.24	0.9	2.02	0.044	1.02-4.92
**ART status**	*on ART	1.47	0.26	2.15	0.032	1.03-2.08
	*not on ART	2.91	0.6	5.15	0	1.94-4.37
	*unknown	2.24	0.9	2.02	0.044	1.02-4.92
**Cotrimoxazole therapy**	*N/A	0.72	0.11	-2.1	0.036	0.53-0.98
	no	1.32	0.38	0.99	0.321	0.76-2.31
**Severe adverse events**	no	0.96	0.13	-0.31	0.756	0.73-1.26
**Co-morbidity**	*Yes	1.63	0.25	3.14	0.002	1.2-2.2

The risk of dying among MDR/RR-TB infected patients who were HIV positive (RRR = 1.57, p = 0.010) is increased by 1.57 times in HIV positive patients while HIV status unknown, the risk was increased by 2.24 times (RRR = 2.24, p = 0.044).

ART status: among TB patients on ART, there seem to be problems with ART monitoring or adherence issues because there were significant deaths occurring among patients on ART as well (RRR = 1.47, p = 0.032).

Co-morbidity: MDR/RR-TB patients with comorbid conditions had increased risk of dying (RRR = 1.63, p = 0.002), comorbidity increased the risk of deaths among MDR/RR-TB patients by 1.63 times.

Table 3 show adjusted RRR after adjusting for other factors included in the multivariate model, the significant factors from multiple binomial regression modelling (Table 3) are listed as follows: Culture conversion period (Not recorded had adjusted RR (RRR = 1.75, p = 0.018); doses missed (≤ 10% missed doses had RRR = 4.75, p < 0.001, > 10% missed doses had RRR = 9.28, p < 0.001, Not recorded had RRR = 3.22, p < 0.001); Comorbidity: TB patients with comorbidities had RRR = 1.44, p = 0.024. Table 4 show results from univariate binomial regression modelling of treatment failure. Age group was significantly associated with treatment outcomes, older ages between 45 to 54 years (RR = 1.65, p = 0.029) and those aged 55+ years (RR = 1.81, p = 0.012) show higher risks of treatment failure respectively. Diagnosis time was associated with treatment outcome, the shorter the time from diagnosis to treatment reduces the chances of treatment failure; the period of 8 to 30 days was protective, reduced the risk of treatment failure by 0.62 times compared to other time intervals. Patents without a recorded culture conversion result had a high risk of treatment failure (RR = 2.96, p < 0.001). The number of treatment doses missed was a predictor of treatment failure, > 10% missed doses (RR = 2.03, p < 0.001). Other factors important were being HIV positive (RR = 1.57, p = 0.01), ART status: HIV+ve on ART (RR=1.47, p=0.032), HIV+ not on ART (RR = 2.91, p < 0.001), HIV status unknown (RR = 2.24, p = 0.044) and comorbidity (RR = 1.63, p = 0.002). Adjusted relative risk ratio after multiple regression modelling show that patient ART status was a significant factor associated with treatment success or failure (ARR = 3.92, p < 0.001). Patients who were HIV positive and not on ART had a high risk of TB treatment failure by 3.92 times compared to patients who were HIV positive on ART treatment (Table 4).

**Table 3:** multiple binomial regression modelling: showing RRR for possible factors of MDR/RR-TB mortality

Death		RRR	Std. Err	Z	P> z	95% Conf. Interval
	25-34	2.85	1.78	1.69	0.092	0.84 - 9.66
	35-44	2.63	1.67	1.52	0.129	0.76 - 9.12
	45-54+	2.16	1.50	1.10	0.270	0.55 - 8.46
	55+	2.82	1.81	1.61	0.107	0.80 - 9.95
	Not recorded	1.37	1.10	0.39	0.696	0.28 - 6.59
**Diagnosis time duration**	8-30 days	1.15	0.40	0.41	0.679	0.58 - 2.28
	31-90 days	0.88	0.37	-0.30	0.765	0.39 - 2.01
	>90 days	1.05	0.48	0.10	0.919	0.43 - 2.55
	Not recorded	0.52	0.25	-1.38	0.166	0.20 - 1.31
*Culture conversion period	> 6 months	0.75	0.57	-0.39	0.699	0.17 - 3.29
	Not recorded	1.75	0.42	2.36	0.018	1.10 - 2.79
*Doses missed	≤10% missed doses	4.75	1.85	3.99	0.000	2.21 - 10.21
	>10% missed doses	9.28	2.04	10.14	0.000	6.03 - 14.27
	Not recorded	3.22	2.88	6.45	0.000	2.40 - 10.75
**HIV status**	HIV+ve	1.00	(omitted)			
	Art status					
	not on ART	1.78	1.21	0.85	0.396	0.47 - 6.74
**Cotrimoxazole therapy**	yes	1.18	0.47	0.42	0.677	0.54 - 2.59
*Comorbidity	Yes	1.44	0.16	-2.25	0.024	0.22 - 0.90

**Table 4:** univariate binomial regression modelling for risk outcomes of treatment failure

Outcome 2		Risk Ratio	Std. Err.	z	P>|z|	95% Conf. Interval
**Sex**	female	0.95	0.11	-0.41	0.685	0.76 - 1.2
*Age group	25-34	1.17	0.24	0.75	0.454	0.78 - 1.76
	35-44	1.24	0.26	1.02	0.306	0.82 - 1.87
	*45-54+	1.65	0.38	2.18	0.029	1.05 - 2.6
	*55+	1.81	0.43	2.5	0.012	1.14 - 2.88
	not recorded	1.08	0.65	0.13	0.9	0.33 - 3.53
**Marital status**	single	1.21	0.17	1.42	0.155	0.93 - 1.58
	widowed	1.36	0.25	1.66	0.097	0.95 - 1.95
	divorced	1.14	0.3	0.49	0.623	0.68 - 1.91
**TB Type**	Rx after LTFU	0.92	0.28	-0.27	0.786	0.5 - 1.69
	Rx after Failure	0.92	0.13	-0.55	0.585	0.7 - 1.22
	Rx/Relapse	0.95	0.15	-0.29	0.774	0.7 - 1.31
*Diagnosis time	*8-30 days	0.62	0.13	-2.3	0.022	0.41 - 0.93
	31-90 days	0.8	0.2	-0.89	0.372	0.49 - 1.31
	>90 days	0.8	0.2	-0.89	0.372	0.49 - 1.31
	not recorded	0.87	0.17	-0.7	0.482	0.59 - 1.28
**Dst resistance - HR**		2.2	0.94	1.83	0.067	0.95 - 5.1
**Dst resistance - ethambutol**		0.8	0.21	-0.85	0.395	0.48 - 1.34
**2.Dst resistance -streptomycin**		0.7	0.2	-1.26	0.206	0.4 - 1.22
*Culture conversion	> 6 months	0.97	0.37	-0.07	0.944	0.46 - 2.05
	*not recorded	2.96	0.38	8.42	0	2.3 - 3.8
*Doses missed	≤ 10% missed doses	1.1	0.23	0.47	0.639	0.73 - 1.67
	*> 10% missed doses	2.03	0.23	6.24	0	1.62 - 2.53
	not recorded	1	(empty)			
**Weight category**	≥50kg	1.04	0.16	0.23	0.822	0.76 - 1.41
*HIV status	*HIV+ve	1.57	0.28	2.57	0.01	1.11 - 2.22
	*HIV unknown	2.24	0.9	2.02	0.044	1.02 - 4.92
*ART status	*not on ART	1.98	0.29	4.75	0	1.49 - 2.63
*HIV/ART status	*HIV+ve, on ART	1.47	0.26	2.15	0.032	1.03 - 2.08
	*HIV+ not on ART	2.91	0.6	5.15	0	1.94 - 4.37
	*HIV status unknown	2.24	0.9	2.02	0.044	1.02 - 4.92
*Cotrimoxazole status	*yes	1.38	0.21	2.1	0.036	1.02 - 1.87
**Serious adverse event**	No	0.96	0.13	-0.31	0.756	0.73 - 1.26
*Comorbidity	*Yes	1.63	0.25	3.14	0.002	1.2 - 2.2

## Discussion

### Statement of principle findings

Patients who missed MDR/RR-TB doses of > 10% were twice more likely to die compared to missing TB doses of = 10%. Patients in age groups 45 - 54 years and those 55 years and above had increased risk of death compared to other age groups.

### Strengths and weaknesses of the study

First, this study included 473 cases of MDR/RR-TB patients under directly observed treatment in a National TB Programme (DOTS), which Strength is notable. In addition, this reasonably big sample size allowed us to control for several variables. Second, this study included patients from all the notifying districts in the country and data was collected for patients initiated on treatment over a five-year period since the country adopted standardized SLDs, hence findings are useful for decision making in a routine programme setting. The study was not without limitations. First, the patients who were referred to rural primary healthcare facilities for follow-up care after MDR-TB treatment initiation were not included. Compared to patients referred, the patients included in our study were more likely to be from urban areas with better socio-economic status, education levels and access to healthcare services.

### Strengths and weaknesses in relation to other studies

In relation to other studies, our study did not report resistance to specific drugs such as INH and /RIF which data is critical in measuring MDR/RR-TB treatment outcomes [24]. Our study also did not report on key variables such as weight, employment status and alcohol abuse which variables may determine treatment success or failure. Our study contrary to other studies recorded data on HIV status of the cohort which variable is critical in measuring TB Treatment outcomes [24]. Our study also reported findings on older age, HIV infection, and extensive TB disease as these are well-known risk factors for mortality of patients with tuberculosis which strengths is notable [25].

### Discussion in important differences in results

In our study, the strongest predictor of mortality was HIV disease especially in patients who were not on ART. Adjusted relative risk ratio from multiple regression modelling show that patient ART status was a significant associated factor of treatment success or failure. Patients who were HIV positive and not on ART had a fourfold risk of death compared to patients who were HIV positive and on ART. This highlights the need for NTP to screen for HIV all MDR/RR-TB patients who become presumptive. This highlights the importance of ART in limiting the unfavourable treatment outcomes especially death among MDR/RR-TB patients in high HIV burden countries. In a study by Kathryn Schnippel *et al*. the strongest predictor of mortality in their cohort was however not HIV infection, but resistance to the most effective second-line drugs in the treatment regimen: preXDR SLID (aRR 1.63), preXDR FLQ (aRR 1.56), or both (XDR-TB, aRR 2.63). This finding however adds weight to recommendations proposing that all DR-TB patients be tested for additional resistance at treatment initiation and highlights the need for new classes of second-line TB treatment [26], In our study, during the study period, Xpert MTB/RIF tests was only reserved for a special category of presumptive TB cases such as re-treatment, Contacts of MDR/RR-TB Patients and the HIV positive patients, among others. In a study by Kathryn Schnippel *et al*. the Xpert MTB/RIF test was not in use in South Africa during the study period and testing for RIF resistance was not universal. The differences in findings however can be attributed to time differences when the said studies were conducted. In our study, the diagnosis of XDR-TB and reported resistance to two effective classes of second-line TB drugs - the fluoroquinolones and second-line injectable drugs was not done. Unlike a study by Ekaterina V *et al*. [27] our study could not determine if these were predictors of mortality during DR-TB treatment and poor rates of treatment success.

### Meaning of the study

First, the period between diagnosis time and initiation of treatment of 8 - 30 days was shown to reduce the risk of TB deaths by 0.62 times compared to longer periods. Failure to initiate patients on MDR/RR-TB treatment early leads to high death rates in addition to having public health ramifications. Second, whilst there was a high uptake of ART among those HIV co-infected, there is need to ensure all MDR/RR-TB patients diagnosed with HIV are timely diagnosed and initiated on ART to lessen the risk of death. Newly diagnosed MDR/RR-TB Patients will benefit the most as they are less likely to know their HIV status upon presentation with presumptive TB hence require special focus. Third, there is need to ensure that patients do not have missed doses during MDR-TB treatment to lessen their risk of death. Continuous and consistent monitoring of missed doses can alert the health care provider to ensure the patient is counselled to enhance adherence and increase the chances of treatment success. Although adherence support by both community and health facility DOTs supporters is commendable to limit the number of missed doses, the monetary incentives then to reduce catastrophic costs was not being implemented during the study period. Forth, much as most patients had a known HIV status in this study, there is great need for all patients to know their HIV status as HIV status which is not known is shown in this study to increase the risk of dying by 2.24 times when compared to those with a known HIV status. It is highly likely however that these patients might have been HIV positive as the study also showed the high death rate in HIV positive ART naïve patients Fifth, consistent monitoring of patients by health care workers or community DOTS monitors to ensure adherence to treatment results in treatment success as missing TB doses of < 10% was shown to result in treatment failure. This will ensure that patients do not miss their doses. The findings also mean that failure to thoroughly screen patients on initiation of treatment for comorbid conditions may result in mortality or treatment failure. These patients have increased risk of dying thus the NTP should therefore ensure a protocol and an algorithm on screening is in place and used. This will ensure that any patient with a comorbid condition is provided with the appropriate treatment and care. Sixth patients in age groups 45 - 54 years and those 55 years and above had increased risk of death compared to other age groups, these results are comparable to findings from a study in South Africa by Kathryn Schnippel *e al*. which also noted that the aged > 60 yrs had an increased risk of dying. This could possibly be due to reduced immunity because of advanced age. The NTP of Zimbabwe and other prorammes in similar settings will therefore need to prioritise patients on care in these age groups to avert possible deaths. Seventh, patients without a recorded culture conversion results had a high risk of treatment failure. Culture conversion results help clinicians to decide the appropriate treatment to be administered to a patient since some patients may require change of treatment regimen based on the culture results. Without culture and DST results, clinicians managed the patients clinically. Lack of availability of culture and DST results has also been a challenge identified in another study by Timire C. *et al*. in a study in Zimbabwe [28]. The Zimbabwe NTP therefore need to ensure CDST results are available for all patients on treatment as per Programmatic Management of Drug Resistance (PMDT) guidelines in Zimbabwe. Lastly, the period between diagnosis time and initiation of treatment of 8 - 30 days was shown to reduce the risk of TB deaths compared to longer periods. The NTP of Zimbabwe and programmes in other similar settings should ensure patients are commenced on MDR/RR-TB treatment as soon as the diagnosis is known. This may entail shortening the turnaround time of results.

### Unanswered questions and future research

It is not known whether missing TB doses of < 10% was intermittent or continuous. Future research is thus required to show the data. It could not also be ascertained which drugs where missed. Future research is required to showcase the data.

### Study limitations

First, non-MDR/RR-TB related deaths such as accidents or other chronic diseases could have been included. However, in accordance with the definition of ‘Death´ In TB patients, any death that occurs during the period of treatment from any cause is attributed to TB. Second, there were missing data on key variables which include CDST results, socioeconomic status, WHO clinical staging, CD4 cell count, nutritional status, MDR-TB drug regimens and their dosages - all which are important factors which may have informed on the predictors of mortality and treatment success. Third, data on co-morbidities was not systematically collected and reported hence, there might have been an underestimation of prevailing comorbidities like diabetes mellitus which require specific diagnostic tests. It is known that patients with comorbidity suffer worse treatment outcomes during treatment.

## Conclusion

Our findings presence a broader view of factors associated with mortality and treatment success in a routine TB programme which factors will add more knowledge and understanding on the part of programme managers and implementers on how to improve programme performance. Factors such as high magnitude of > 10% missed doses, poor monitoring of patients due to incomplete documentation, prevalent comorbidities, missed ART opportunities i.e. Patients who had an unknown HIV status but could have been HIV positive had a higher risk of death, Improving ART uptake among those ART-naïve and strategies aimed at improving treatment adherence are important in improving treatment success rates and avert death. On the other hand, being not on ART when HIV positive was a major significant predictor of mortality. Future studies should focus on profiling management of MDR/RR-TB patients accessing care at the primary level health care facilities in this setting.

### What is known about this topic

- High death rate in HIV positive patients not on ART receiving DR/RR-TB treatment;
- ART improves treatment success rates in MDR/RR-TB patients with TB/HIV co-infection;
- High rates of HIV infection in MDR/RR-TB patients.

### What this study adds

- Thorough screening of MDR/RR-TB patients for comorbidities at initiation of treatment helps reduce the risk of treatment failure;
- Consistent monitoring of patients to ensure adherence to treatment reduces the risk of treatment failure;
- Early diagnosis and initiation of treatment reduces the risk of MDR/RR- TB deaths compared to longer periods.
